# Impact of Commercial Strain Use on *Saccharomyces cerevisiae* Population Structure and Dynamics in Pinot Noir Vineyards and Spontaneous Fermentations of a Canadian Winery

**DOI:** 10.1371/journal.pone.0160259

**Published:** 2016-08-23

**Authors:** Jonathan T. Martiniuk, Braydon Pacheco, Gordon Russell, Stephanie Tong, Ian Backstrom, Vivien Measday

**Affiliations:** Wine Research Centre, University of British Columbia, Vancouver, British Columbia, Canada; University of Strasbourg, FRANCE

## Abstract

Wine is produced by one of two methods: inoculated fermentation, where a commercially-produced, single *Saccharomyces cerevisiae* (*S*. *cerevisiae*) yeast strain is used; or the traditional spontaneous fermentation, where yeast present on grape and winery surfaces carry out the fermentative process. Spontaneous fermentations are characterized by a diverse succession of yeast, ending with one or multiple strains of *S*. *cerevisiae* dominating the fermentation. In wineries using both fermentation methods, commercial strains may dominate spontaneous fermentations. We elucidate the impact of the winery environment and commercial strain use on *S*. *cerevisiae* population structure in spontaneous fermentations over two vintages by comparing *S*. *cerevisiae* populations in aseptically fermented grapes from a Canadian Pinot Noir vineyard to *S*. *cerevisiae* populations in winery-conducted fermentations of grapes from the same vineyard. We also characterize the vineyard-associated *S*. *cerevisiae* populations in two other geographically separate Pinot Noir vineyards farmed by the same winery. Winery fermentations were not dominated by commercial strains, but by a diverse number of strains with genotypes similar to commercial strains, suggesting that a population of *S*. *cerevisiae* derived from commercial strains is resident in the winery. Commercial and commercial-related yeast were also identified in the three vineyards examined, although at a lower frequency. There is low genetic differentiation and *S*. *cerevisiae* population structure between vineyards and between the vineyard and winery that persisted over both vintages, indicating commercial yeast are a driver of *S*. *cerevisiae* population structure. We also have evidence of distinct and persistent populations of winery and vineyard-associated *S*. *cerevisiae* populations unrelated to commercial strains. This study is the first to characterize *S*. *cerevisiae* populations in Canadian vineyards.

## Introduction

Wine fermentation—the conversion of grape must sugars to ethanol—is conducted by one of two methods: inoculated fermentation, where a commercially-produced, single *S*. *cerevisiae* yeast strain is used; or the traditional spontaneous fermentation, where yeast present on grape and winery surfaces carry out the fermentative process [1]. Spontaneous fermentation is characterized by a diverse succession of yeast genera, species and strains, with either one or multiple strains of the more ethanol-tolerant *S*. *cerevisiae* dominating the final stages [2–5]. As a result, spontaneously-fermented wines may be more organoleptically complex than commercial strain-fermented wines due to the contribution of a greater range of metabolic byproducts from the different yeasts [4,6,7]. It is also widely believed that spontaneous fermentation may impart more regional character to wines due to region-specific yeast populations [4,8]. Inoculated fermentation, however, is the method of choice in North America and in other global winemaking regions as it produces reliable results consistent from year to year [9]. Some wineries use both fermentation methods, but in these facilities, commercial yeast may dominate spontaneous fermentations. A number of studies examining *S*. *cerevisiae* strain populations in spontaneous fermentations conducted in such facilities have found that commercial strains are present in and may dominate these fermentations [10–17]. Commercial yeast strains have also been shown to disseminate into the vineyard from winery facilities but they do not persist over multiple vintages [18,19].

The influence of geography on *S*. *cerevisiae* population structure has been examined on a wide range of scales worldwide [18,20–26]. While ecological niche appears to play a larger role than geographical origin in defining *S*. *cerevisiae* population structure on a global scale [27–29], geography appears to be a significant driver of structure within wine- and vineyard-associated *S*. *cerevisiae* populations as found in New Zealand [21,23,26] and Portugal [20,24]. These studies, however, have examined yeast communities in regions that range from 10km to 1000km in distance. A variety of methods, including mitochondrial DNA restriction fragment length polymorphism (mtDNA-RFLP), inter-delta PCR [30] and more recently, SNP analysis [28,29] and RAD-seq [31] have been used to discriminate between *S*. *cerevisiae* strains and elucidate strain population structure in winemaking regions and even across populations globally. Microsatellite analysis, however, remains a popular and lower-cost method easily tailored to high-throughput analysis and is capable of high discrimination between *S*. *cerevisiae* strains, having been used continually over the last 15 years in many wine and vineyard-associated *S*. *cerevisiae* studies [18,21–24,26,32,33].

To date, little research has been published on *S*. *cerevisiae* population dynamics in spontaneous fermentations in Canada [14,16], and no study on Canadian vineyard-associated *S*. *cerevisiae* strains has been conducted. Additionally, while *S*. *cerevisiae* populations in spontaneous fermentations and in vineyards have been thoroughly profiled in various wine regions [e.g. 17,20,23,25,33–35], focus has mostly remained on either vineyard or winery populations, but rarely both. No research has directly addressed how the winery environment and use of commercial *S*. *cerevisiae* strains influences the vineyard *S*. *cerevisiae* population composition prior to harvesting and winery processing, which we undertake for the first time in this study. We collaborated during the 2013 and 2014 vintages with a winery in British Columbia (BC), Canada that has conducted both inoculated and spontaneous fermentations for several years. To determine the impact of concurrent commercial strain use in the winery on spontaneous fermentation *S*. *cerevisiae* strain composition, we conducted spontaneous fermentations of grape samples from one of the winery’s Pinot Noir vineyards and compared the populations to those in winery spontaneous fermentations of grapes from the same vineyard. We also profiled *S*. *cerevisiae* populations in two other closely situated Pinot Noir vineyards managed by the same winery in the same manner. Lastly, to elucidate the population dynamics of *S*. *cerevisiae* during spontaneous fermentation, we compared *S*. *cerevisiae* population composition between early, mid and late stages of all fermentations. We classified *S*. *cerevisiae* strains by analysis of 8 polymorphic microsatellite loci and developed a database of microsatellite patterns for 72 commercial strains. Our results revealed that the winery environment significantly influences the *S*. *cerevisiae* population composition in spontaneous fermentations due to use of commercial strains. Interestingly, winery spontaneous Pinot Noir fermentations were not dominated solely by commercial strains, but by strains with genotypes similar to commercial strains, suggesting a population of *S*. *cerevisiae* derived from commercial strains is resident in the winery. We did not find significant genetic differentiation or variation in strain richness between yeast populations at different fermentation stages. There is low but significant genetic differentiation between *S*. *cerevisiae* populations in the vineyards and the winery, and commercial yeast appear to be an important driver of this population structure. While commercial and commercial-related strains were identified in all vineyards, we also found evidence of distinct *S*. *cerevisiae* sub-populations unrelated to commercial strains that were present in both vintages. This study is the first to profile and identify *S*. *cerevisiae* populations in a Canadian vineyard.

## Methods

### Vineyard (Lab) and winery spontaneous fermentations

**Fig 1:**
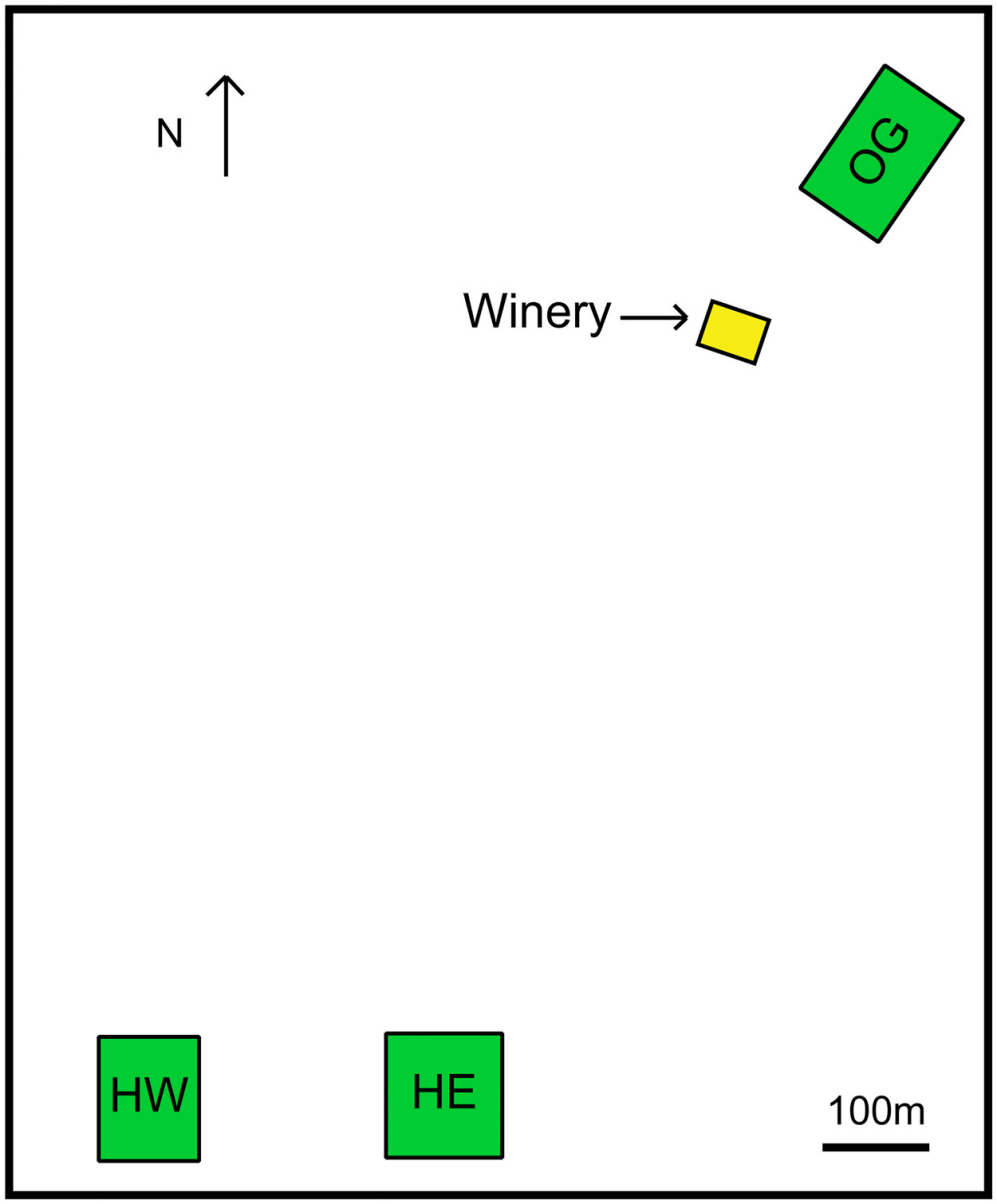
Map of relative vineyard and winery locations. The vineyard blocks and distances are drawn to scale.

Stoneboat Vineyards (SBV) gave access to their Home East (HE), Home West (HW) and Orchard Grove (OG) Pinot Noir vineyard blocks for the purposes of our study (Fig 1). Over a week in autumn 2013 and 2014, we aseptically harvested healthy grape clusters from each block within a week prior to the winery harvest date. Three ~7-10kg samples were harvested from each vineyard block. Six 60-foot row sections (three 20-foot post panels, each containing five to eight grapevines), evenly distributed across each vineyard block, were selected for sampling. Outer rows were excluded. One post panel per row section was assigned to a sample, and six randomly selected clusters were harvested per panel (three clusters from the east side of the row, three from the west). The grapes were never introduced into the winery but taken directly to the laboratory on ice. All samples were processed within 7 hours of harvesting. Each 10kg sample was destemmed, crushed and fermented aseptically (including skins) in separate 3L sterile, airlock-sealed vessels, for a total of 9 fermentations (3/vineyard). Fermentations were conducted at 25°C and sampled after crushing and at early, mid and late fermentation (0%, ~30%, ~50%, ~90% sugar depletion as determined by weight). Samples were plated in duplicate serial dilutions on YPD containing 150mg biphenyl + 100mg chloramphenicol to inhibit mold and bacterial growth, respectively [36].

No later than one week after vineyard sampling in 2013–14, Pinot Noir grapes were harvested by the winery from OG block for industrial fermentations. Three 500L fermentations were conducted by the winery in sanitized plastic vats, labelled B1, B2, and B3. To carry out each fermentation, three 500 kg bins of grapes were selected from across different areas of the OG vineyard, destemmed and crushed into a vat, heated to maintain a temperature of 25°C and covered. No sulfur or fermentation nutrients were added. The fermentation “caps” of skins were submerged daily and before sampling. Any tools used to manage or sample the fermentations were sanitized and wiped with 70% ethanol before use between fermentations. Sampling points were determined by density measurement with a hydrometer in degrees Brix and correspond to the equivalent sugar depletion levels listed above. Wine samples were shipped on ice overnight to the lab for plating.

### *S*. *cerevisiae* isolation & DNA extraction

Where possible, up to 32 *Saccharomyces* colonies were isolated at each fermentation sampling point from plates containing 30–300 *Saccharomyces* colonies. (In some instances, *Saccharomyces* were present in lower numbers or were absent from early-stage fermentations. No *Saccharomyces* were detected in t = 0 samples). Isolates were confirmed as *Saccharomyces* by plating on Wallerstein Agar, which differentiates *Saccharomyces* based on colony colour [37], and by negative growth on Lysine Agar [38]. All isolates were arrayed in 96-well tissue-culture plates and frozen at -80°C for later use. Yeast DNA was extracted from single colonies using a modified procedure [39] where a 1.25mg/mL Zymolyase digest was performed on a small yeast colony for 30min at 37°C followed by incubation at 95C for 10min. The yeast lysate was centrifuged at 4000 *x g* for 10min to pellet cell debris and the supernatant was removed and diluted for use in PCR.

### Microsatellite analysis

We selected 8 short sequence repeat (SSR) loci (see Table 1) from a set of 10 compiled by Richards et al. 2009 [40] and amplified these loci according to their protocol, substituting the HEX 5’fluorescent primer tag with VIC dye. We also amplified fragments of the MATa and MATα loci in all isolates, as their lengths can distinguish between *S*. *cerevisiae* and some other *Saccharomyces* species [32]. Amplicons were measured by capillary electrophoresis with an AB3730 DNA Analyzer at the UBC Nucleic Acids and Proteins Unit. Peak calling was performed in STRand [41] and allele binning in MsatAllele [42] to create multi-locus genotypes (MLGs). Each unique MLG was considered as a separate strain. Isolates that did not amplify or only partially amplified were re-analyzed and eliminated from the dataset upon a second amplification failure. To identify commercial *S*. *cerevisiae* isolates, we typed 72 commercial strains including all strains ever used by the winery. We also referenced the commercial strain database from Richards et al. 2009 [40] to screen for an additional ~40 strains, allowing for a ±1bp difference between matching database and isolate allele sizes to account for differences in analysis binning methods.

**Table 1:** Microsatellite loci used in this study, selected from Richards et al. 2009.

Locus	SSR	CHR	Tag	Dir	Primer Sequence
*YGL139W*	CAA	VII	FAM	F	GTGTCTCTTTTTATTTACGAGCGGGCCAT
				R	AAATCTCATGCCTGTGAGGGGTAT
*YFR028C*	GT	VI	VIC	F	GTGTCTTGACACAATAGCAATGGCCTTCA
				R	GCAAGCGACTAGAACAACAATCACA
*YGL014W*	TAA	VII	NED	F	GTGTCTCAGGTCGTTCTAACGTTGGTAAAATG
				R	GCTGTTGCTGTTGGTAGCATTACTGT
*YOL109W*	TAA+TAG	XV	VIC	F	GTGTCTAGGAGAAAAATGCTGTTTATTCTGACC
				R	TTTTCCTCCGGGACGTGAAATA
*YML091C*	AAT	XIII	NED	F	GTGTCTAAGCCTCTTCAAGCATGAC
				R	GTGTCTGGACAATTTTGCCACCTTA
*YLL049W*	TA	XII	FAM	F	GCAACATAATGATTTTGAGGT
				R	GTGTCTTGTGTGAGCATAGTGGAGAA
*YDR160W*	AAT	IV	NED	F	GTGTCTGAGGAGGGAAATGGACAG
				R	GCCTGAAGATGCTTTTAG
*YPL009C*	CTT	XVI	FAM	F	GTGTCTGGGTTTTGGATTTTTATGGA
				R	GTGTCTTTCAATTTTCCTCTTTTACCAC
*MAT*α	-	III	FAM	F	CAGCACGGAATATGGGACT
*MATa*	-	III	VIC	F	CAATGATTAAAATAGCATAGTCGG
				R	GGTGCATTTGTCATCCGTC

SSR, short sequence repeat; CHR, chromosome; Dir, primer direction.

### Data analysis

Microsatellite data manipulation, genotype accumulation curves and Bruvo genetic distance calculations were performed in R (v3.2.5) [43] in the package Poppr 2.1.1 [44]. Rarefaction was calculated using vegan 2.3–1 [45] with a minimum common sample size of 15 for sample points and 56 for fermentations [46]. Basic population information and F-statistics were calculated in GenAlEx 6.5 [47,48]. In order to reduce clonal bias, these analyses were performed on datasets filtered for duplicate genotypes (clone-censored) by vineyard/winery source. Venn diagrams were modelled in jvenn [49]. To assess population structure, we performed Bayesian clustering analysis on the combined 2013 and 2014 clone-censored datasets in InStruct [50] using the admixture model with a burn-in of 50 000 iterations and a total run of 100 000 iterations with 5 chains per cluster, or K, from K = 4 to K = 30. The analysis was rerun for K = 9 to K = 20 with 10 chains of 1 000 000 iterations per K and a burn-in of 100 000. The optimal number of subpopulations was determined using the Deviance Information Criterion method outlined in Gao et al. (2011) [51]. InStruct chains were aligned in CLUMPP using the *LargeKGreedy* algorithm, with 300 000 random input orders [52]. The CLUMPP output was visualized in DISTRUCT [53]. The relationship between *S*. *cerevisiae* population structure and location of origin was quantified using ObStruct [54]. T-tests, Friedman test, Pearson’s Chi-square and *post-hoc* tests were performed in SPSS 24 [55]. The phylogenetic network was visualized in SplitsTree 4.0 [56].

## Results

### Prevalence, distribution and abundance of commercial and other strain genotypes

SBV is a winery situated in the Okanagan Valley of BC, Canada. The region is considered cool-climate as it has a fairly short growing season (April to October). In autumn of 2013 and 2014, triplicate Pinot Noir grape samples were collected from each of SBV’s three geographically separate Pinot Noir vineyards (HE, HW, & OG, Fig 1) and were crushed and fermented aseptically in the lab. Separate fermentations were carried out in triplicate for each vineyard and all grape samples successfully fermented. Simultaneously, triplicate large-scale spontaneous fermentations of OG vineyard grapes were conducted at SBV. Samples were plated from both lab and winery fermentations at t = 0 (crushing), early, mid and late stage fermentations on selective yeast media for single *S*. *cerevisiae* colonies, for a total of 84 individual fermentation sample points. Colonies were confirmed as *Saccharomyces* by plating on Wallerstein and Lysine Agars. A total of 1986 *S*. *cerevisiae* isolates were collected from the 2013 and 2014 vintages and 1927 produced complete microsatellite profiles across the 8 selected loci, 870 from 2013 and 1057 from 2014. No *Saccharomyces* were detected in t = 0 samples. From the 1927 *S*. *cerevisiae* isolates, 254 different MLGs were identified (S1 Table). Rarefaction analysis performed in Poppr indicated that for all sampling points yielding 15 or more *S*. *cerevisiae* isolates, the number of isolates analyzed sufficiently represented *S*. *cerevisiae* MLG richness at that sampling time in the fermentation [46]. S2 Table lists the total number of isolates, MLGs, and expected MLGs from each fermentation sample point. A genotype accumulation curve was implemented to test the discriminatory power of our eight-locus microsatellite. The curve had a slight plateau which indicated that the number of loci is sufficient to capture most (>90%) of the MLGs present (S1 Fig).

Thirteen of the 254 MLGs identified in our isolate collection were identical to commercial yeast MLGs. Many more MLGs were highly similar to commercial genotypes, some with heterozygous mutations at certain loci, but most with loss of heterozygosity at one or more loci. To further examine the relatedness of MLGs isolates to commercial strains, we used Bruvo’s distance, which accounts for stepwise mutations in microsatellites [57]. When MLG minimum distances to commercial strains are plotted by frequency, a bimodal distribution of distances is evident, with a high number of MLGs either closely or distantly related to commercial strains (S2 Fig). We considered all strains falling below a minimum Bruvo’s distance of 0.25 to be related to commercial strains. In total, 102 strains were classified as commercial-related, while the remaining 139 MLGs were considered unique genotypes.

**Fig 2:**
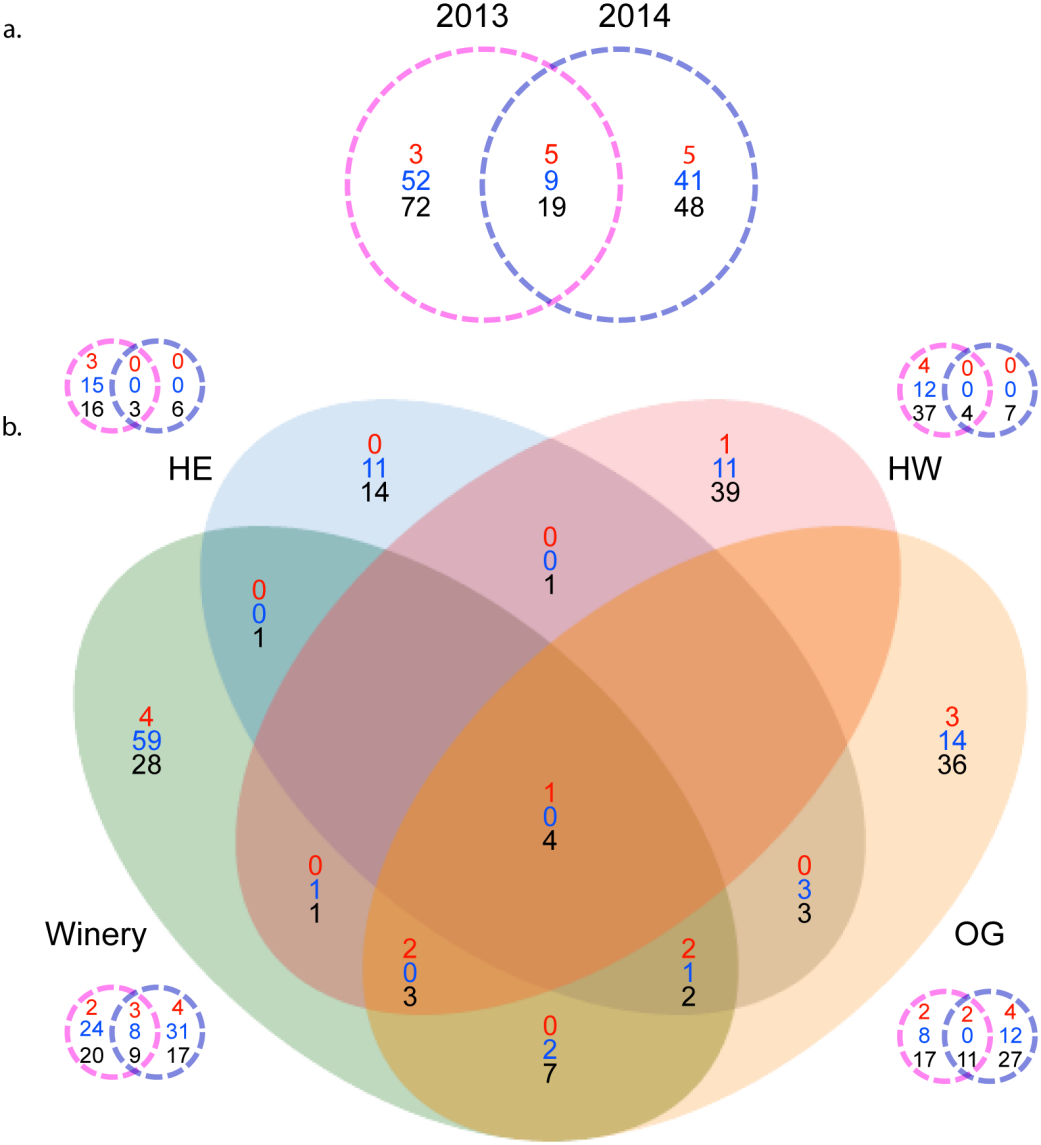
Venn diagrams of MLG distribution by vintage and location. Red numbers, commercial MLGs; blue numbers, commercial related MLGs; black numbers, unique MLGs. a) Venn diagram of strain distribution by vintage. b) Venn diagram of MLG distribution between vineyard and winery locations. Venn diagrams adjacent to the main diagram display the MLG distribution for each location by vintage.

A comparison of the microsatellite data across vintages and location revealed that the majority of strains are geographically and temporally distinct (Fig 2). Only 33 of 254 genotypes were identified in both vintages, and only 5 (Commercial strain Lalvin ICV D254^®^; unique strains SBV014, 015, 016 and 050) were isolated from all 3 vineyard sites and the winery fermentations. Of these, only 2 (SBV014, 015) were isolated from both vintages. The winery had the highest number of MLGs shared with other locations, in particular with the OG vineyard (Fig 1), where the grapes were sourced for these fermentations. There were fewer strains shared between vineyard locations, and no commercial-related strains were isolated from the vineyards in both years. The total number of MLGs identified per year dropped from 160 in 2013 to 127 in 2014 (Fig 2a). Commercial and commercial-related MLGs were ubiquitous in all winery and OG fermentation sets from both vintages. Interestingly, although commercial and commercial-related genotypes were present in the 2013 HE and HW fermentations, none were identified in the 2014 HE and HW fermentations. All commercial MLGs and commercial-related MLGs (which are labelled “SB” followed by the related commercial strain) found in each location and vintage are presented in S3 Table. As indicated by an asterisk, four commercial strains were identified (Lalvin BA11^®^, Lalvin RA17^®^, Lalvin Rhone 2056^®^, Uvaferm SVG) that the winery has never used. In addition, the winery has never used the parent strains of several commercial-related MLGs we detected, such as SB_522 Davis and SB_Anchor VIN7 (indicated by a number sign, S3 Table). We identified other MLGs related to strains used by the winery (e.g. SB_Laffort X5), but not their parental genotypes (indicated by a dagger, S3 Table). The winery used all remaining commercial yeast genotypes for other fermentations in both vintages sampled and in previous years, although some of these commercial yeasts were not used until after our experimental winery fermentations were complete.

**Fig 3:**
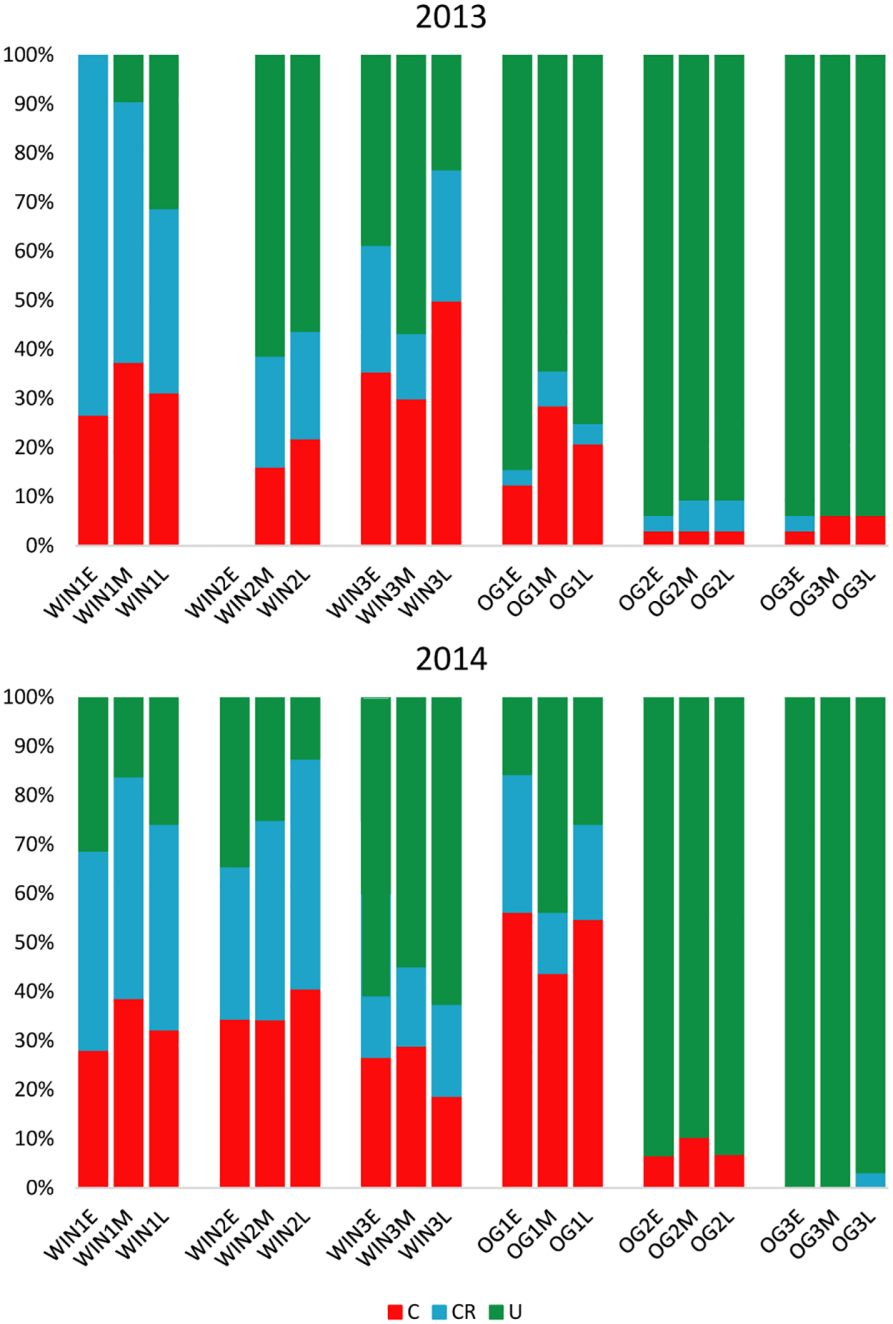
MLG type frequency charts for 2013 and 2014 winery and OG vineyard fermentations. Columns labeled WIN represent winery fermentations; columns labeled OG represent OG vineyard fermentations. Each 3-column cluster represents a single fermentation out of triplicate fermentations (numbered 1, 2, 3). The colors in each column [red, commercial (C); blue, commercial-related (CR); green, unique (U)] represent the frequencies of MLG types at a fermentation stage (E, early; M, mid; L, late). The early timepoint of the second winery replicate in 2013 was omitted as <10 isolates were collected.

To investigate whether the number of commercial MLGs was significantly higher in the winery than in the OG vineyard, we performed a Chi-square test of independence on a table of MLG types from both locations (Table 2). While there is no significant difference between the expected count and observed count of commercial MLGs in either the winery or OG, the winery commercial-related MLG count is roughly three times higher. Correspondingly, the winery unique MLG count is lower than expected. We also performed the same test on MLG types from all vineyards and found no significant deviation from the expected values (χ^2^ = 4.428, p = 0.352). As a means of assessing the degree of commercial strain dominance in winery versus OG vineyard fermentations, we calculated the average relative abundance of commercial and commercial-related MLGs at late fermentation in these locations (Table 3). The values are averages of 2013 and 2014 fermentations from each site (n = 6), except for OG commercial MLG abundance, where one outlier (= 55%) was removed. We found that the proportions of both commercial and commercial-related MLGs in the total late fermentation were roughly equivalent to each other in both the winery and vineyard locations. Both MLG types were significantly more abundant in winery fermentations than in OG fermentations. The ratios of MLG types in OG and winery fermentations are depicted in frequency charts (Fig 3).

**Table 2:** Chi-square test of independence between OG vineyard/winery and type of *S*. *cerevisiae* MLG.

Location	C	CR	U
**Winery**	**9** (-0.50)	**63**^a^ (4.15)	**46**^b^ (-3.81)
	9.98	48.73	59.29
**OG**	**8** (0.50)	**20**^b^ (-4.15)	**55**^a^ (3.81)
	7.02	34.27	41.71

**χ**^**2**^ = 17.576, p ≤ 0.00015

U, unique; CR, commercial-related; C, commercial. Yeast MLG counts are in bold with adjusted standardized residuals (ASR) in parentheses. Expected values are listed below. Counts are censored by vineyard only and include both 2013 and 2014 vintages.

^**a,b**^ indicate counts significantly higher and lower than expected results, respectively; p < 0.008 (Bonferroni-adjusted p-value). Significance was determined by squaring ASRs and calculating P_obs_>P_random_ from the χ^2^ distribution.

**Table 3:** Proportional abundance (%) of commercial and commercial-related MLGs in late-stage fermentations from the winery and OG vineyard.

Location	C (%)	CR (%)
Winery	31.9±10.7*	32.0 ± 11.6*
OG	6.4±5.9%*†	5.3 ± 7.3 *

C = commercial yeast MLGs, CR = commercial-related yeast MLGs. C and CR values labelled with * differ significantly at p < 0.001 as determined by Student’s T-test assuming unequal variance.

^†^Outlier value removed (C = 55%). All other values are the average of six (2013 = 3, 2014 = 3) fermentations from the winery and OG vineyard.

We isolated *S*. *cerevisiae* from all vineyard and winery fermentations at early, mid, and late stages to determine whether there were changes in yeast population composition during the course of fermentation. Calculation of pairwise fixation indices (F_ST_) revealed extremely low and insignificant genetic differentiation between *S*. *cerevisiae* populations at different timepoints (Table 4). A Friedman test of repeated measures performed on the rarified MLG richness values from all fermentations across all stages found no significant change in strain richness over time (χ^2^ = 1.200, p = 0.573).

**Table 4:** Pairwise fixation index (F_ST_) values for *S*. *cerevisiae* populations at different stages of fermentation.

	**Early**	**Mid**	**Late**
**Early**	—	0.273	0.133
**Mid**	0.004	—	0.946
**Late**	0.005	0.001	—

F_ST_ values are below the diagonal with p-values above the diagonal.

### Winery and vineyard *S*. *cerevisiae* population structure and genetic differentiation

*S*. *cerevisiae* winery and vineyard population observed heterozygosity (H_o_) were approximately two to four times lower than expected (Table 5), and all populations deviated significantly from Hardy-Weinberg equilibrium (p < 0.0001) at all loci. F_IS_ values indicate all populations are inbred, which is typical of the budding yeast in nature [58]. Allele counts, observed and expected heterozygosity, and inbreeding coefficient values by locus can be found in S4 Table. To estimate the genetic differentiation between *S*. *cerevisiae* populations from the winery and vineyards, we calculated pairwise fixation indices (F_ST_). MLGs were clone-censored by vineyard prior to analysis. Based on the F_ST_ values and their respective probabilities, we found significant but low differentiation *S*. *cerevisiae* populations between most locations (Table 6). The largest difference was between the winery and HW with an F_ST_ value of 0.053. No significant genetic differentiation was found between OG and HE vineyard populations. We also evaluated the genetic differentiation between locations after removing commercial and commercial-related genotypes from the datasets. Removal of commercial and commercial-related genotypes caused a decrease in nearly all F_ST_ values, and the level of genetic differentiation between the winery and HE became insignificant (p > 0.05).

**Table 5:** Summary of population information for winery and vineyard *S*. *cerevisiae* populations.

	Winery	HE	HW	OG
**No. alleles**	15±2	9±1	10±1	14±2
**H**_**o**_	0.443±0.22	0.328±0.034	0.186±0.029	0.291±0.025
**H**_**e**_	0.811+0.025	0.817+0.016	0.732±0.032	0.81±0.020
**F**_**IS**_	0.45±0.032	0.599±0.039	0.743±0.042	0.641±0.029

Values are the average of each measure across eight loci ± standard error. H_o_ = observed heterozygosity, H_e_ = expected heterozygosity, F_IS_ = inbreeding coefficient.

**Table 6:** Pairwise F_ST_ values for *S*. *cerevisiae* populations from different locations, with and without commercial and commercial-related genotypes.

	With C & CR strains				Without C & CR strains			
	Winery	HE	HW	OG	Winery	HE	HW	OG
**Winery**	—	0.0003	0.0001	0.0001	—	0.190	0.002	0.011
**HE**	0.028	—	0.0004	0.0787	0.018	—	0.036	0.263
**HW**	0.053	0.031	—	0.0020	0.029	0.026	—	0.050
**OG**	0.026	0.011	0.016	—	0.021	0.016	0.015	—

F_ST_ values are below the diagonal with p-values above the diagonal. C, commercial MLGs; CR, commercial-related MLGs.

**Fig 4:**
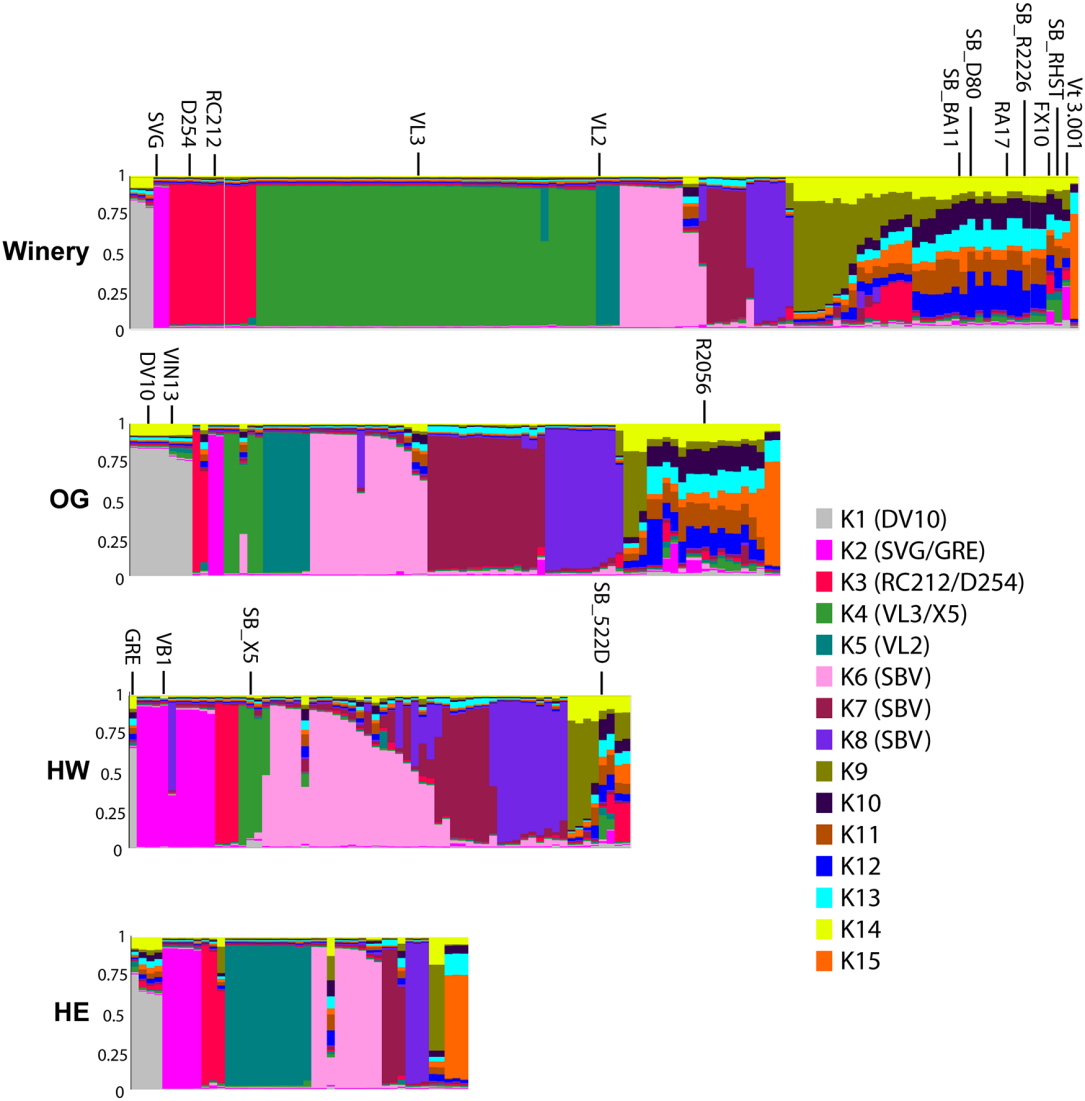
DISTRUCT plots of inferred ancestry profiles from vineyard and winery *S*. *cerevisiae* populations. Each column represents an ancestry profile for an individual MLG. Each colour corresponds to a subpopulation/cluster. The scale of each colour in an ancestry profile represents the proportion (membership coefficient) of the individual’s ancestry profile assigned to that cluster, as measured on the y-axis of each plot. The legend indicates the subpopulation/cluster number and colour. An ancestry profile for each commercial and commercial-related MLG in the dataset is noted in the legend. Subpopulations containing commercial MLGs with membership coefficients >80% are labelled with the strain name in parentheses, unique subpopulations with SBV in parentheses.

To further analyze population structure in our dataset independent of location, we used the Bayesian clustering software InStruct. InStruct does not assume Hardy-Weinberg equilibrium, but instead assigns individuals proportional membership coefficients in different subpopulations based on inbreeding rates, which is well suited for *S*. *cerevisiae* population analysis [50]. We ran InStruct on the MLG dataset clone-censored by vineyard/winery, identifying an optimal K of 15 subpopulations or clusters. CLUMPP alignment of 10 InStruct runs yielded an H of 0.88, indicating high similarity between replicate runs. The DISTRUCT plots depict the CLUMPP-aligned inferred ancestry profiles for each individual MLG, grouped by vineyard (Fig 4). Each column in the plot represents an individual MLG, while each colour represents an inferred subpopulation. Individuals whose columns consist of a single color belong to a single subpopulation, while individuals with columns consisting of two or more colours are the result of interbreeding, or admixture, between the subpopulations. We considered individuals with membership coefficients >0.80 in a particular cluster to belong to that subpopulation. Five subpopulations consist primarily of commercial and commercial-related member genotypes (K1-K5, Fig 4). Certain commercial strains were grouped in the same cluster (e.g. Lalvin RC212^®^ and D254^®^ in K3). All commercial-related genotypes were grouped with their parent strains, agreeing with our *ad hoc* MLG classifications defined earlier; however, some genotypes classified as unique were also grouped in these clusters, indicating they are related to commercial yeast. Interestingly, three subpopulations (K6-8, labelled SBV in Fig 4) are not associated with commercial genotypes. These three subpopulations collectively have 60 genotypes with membership coefficients >0.80 (see S6 Table for a list of MLGs). There is a noticeable amount of admixture between these clusters in the vineyards (e.g. see K6-8 in HW plot, Fig 4). The remaining seven clusters have no individuals with membership coefficients > 0.80 (K9-15); of these, K10-14 have no individuals with more than 30% membership in any cluster, also indicating significant admixture between subpopulations. The ancestry profiles of several commercial MLGs in the dataset are divided across several of these clusters (e.g. FX10, Vt3.001 in winery plot, Fig 4), which may indicate their descent from multiple ancestral subpopulations. The winery shows the highest level of diversity as all 15 clusters are represented in the plot. There is a noticeably higher number of individuals in commercial subpopulations (K1-5) in the winery versus the OG vineyard, which supports the Chi-square test results. The vineyards have a higher number of individuals in unique MLG-associated clusters (K6-8).

We further related population structure to location by analyzing the Bayesian clustering results in Obstruct, which quantifies the relationship between Bayesian-inferred population structure and a factor of interest with a correlative statistic, R^2^. ObStruct results are included in S5 Table. *S*. *cerevisiae* population structure is significantly correlated with vineyard and winery location (R^2^ = 0.07, p < 0.0001). Pairwise R^2^ values between locations agree well with pairwise F_ST_ results in magnitude; e.g. fixation index and R^2^ values between the winery and HW are both largest (F_ST_ = 0.053, R^2^ = 0.07). MLGs from the winery and HW were the largest contributors to population structure. Removal of winery MLGs caused the R^2^ value to drop to 0.04, removal of HW MLGs to 0.06. Removal of the HE population did not affect the R^2^ value, while removal of the OG MLGs caused a slight increase, indicating its population is less structured relative to the other locations. Notably, when the inferred VL3 cluster (K4) is removed from the dataset, the overall R^2^ value declined to 0.05. The R^2^ value was unaffected when any of the other clusters were removed.

**Fig 5:**
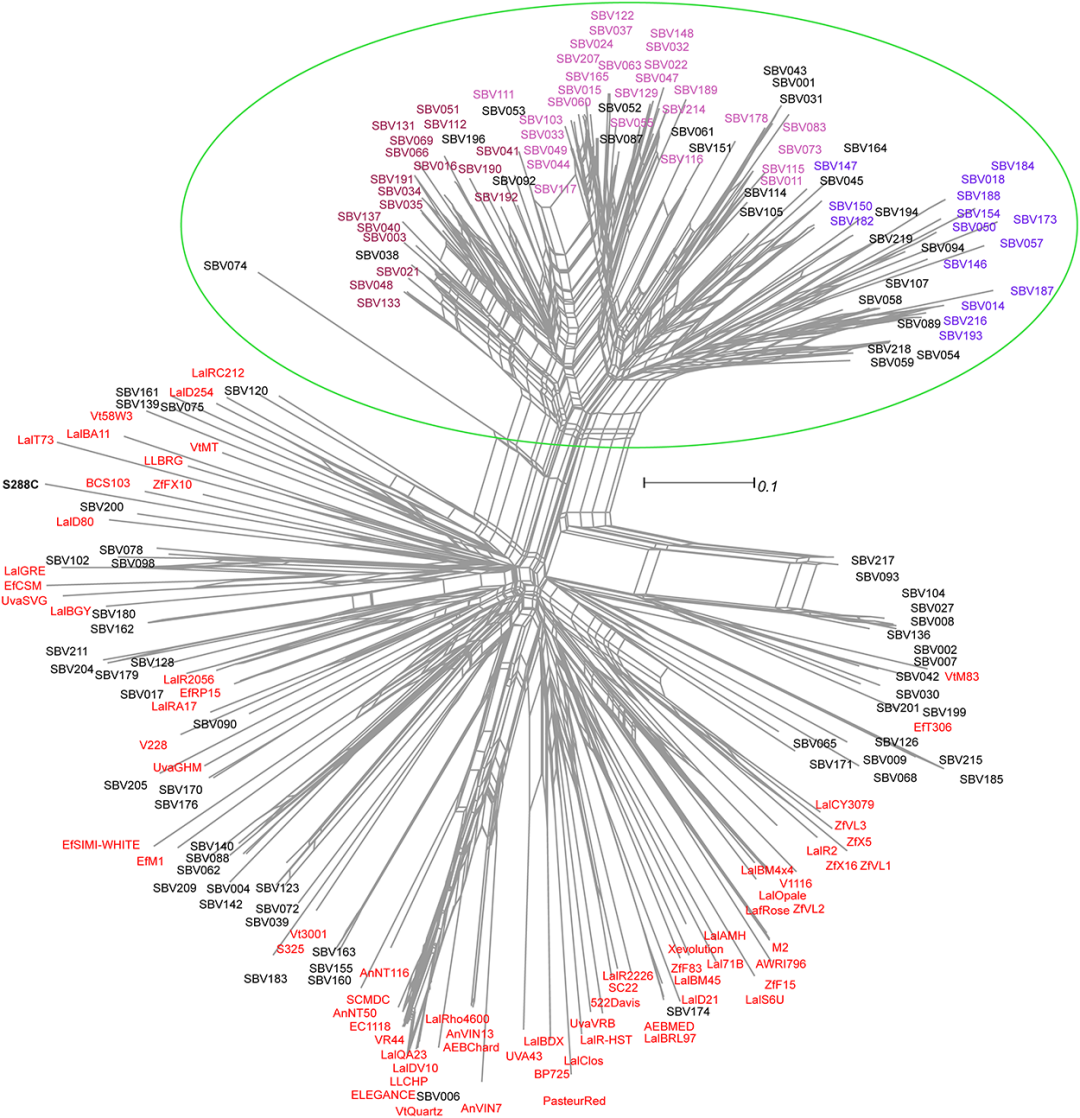
Phylogenetic network of database commercial yeast and unique vineyard/winery MLGs. An unrooted phylogenetic network computed from a Bruvo Distance matrix using the Neighbor-Net method [59] and drawn to scale. Unique strains are labelled “SBV” followed by a unique number. Commercial strains are in red. Isolates with > 80% membership in one of three unique subpopulations (K6-K8) are colored by cluster as in Fig 4.

We constructed a phylogenetic network that includes all our commercial genotypes and unidentified MLGs in our dataset to produce a better picture of the relationships between these strains (Fig 5). We excluded commercial-related MLGs to reduce the network size. While some of the unique MLGs (labelled as ‘SBV’) in our dataset are clearly related to commercial strains, there is a separate cluster consisting only of unique strains (Fig 5, circled in green), which suggests that these MLGS represent a distinct group of yeasts in the vineyard and winery that are unrelated to commercial strains. Many of the MLGs in this cluster have membership coefficients >0.80 in one of the three unique subpopulations (K6-8) and are labelled by colour. Comparison of the cluster K6-8 strains in the network to the commercial MLG database revealed that nearly all individuals differ from every commercial MLG by at least 10 out of 16 alleles (one individual differed by 9/16 alleles) with a minimum Bruvo distance to commercial strains greater than 0.43. Notably, the two yeast MLGs identified in all locations and both vintages (SBV014, 015) are part of this distinct group.

## Discussion

### Commercial yeast in the winery and vineyards

Wine and vineyard associated *S*. *cerevisiae* populations have been widely researched over the last 20 years. While not universal [12,23,33], the potential for introduction of commercial yeast into spontaneous fermentations is well documented in various wine regions [10–16]. Most studies examining this phenomenon, however, have identified the *S*. *cerevisiae* populations in winery fermentations, and have not examined the grape-associated vineyard populations prior to contact with winery equipment, while others have not included replication or sampling over multiple vintages. By surveying *S*. *cerevisiae* populations in the vineyard through multiple samplings over two vintages and comparing these to populations in multiple fermentations of the same grapes after winery processing, we have examined how the winery environment and commercial strain use influence the composition of the initial grape-associated *S*. *cerevisiae* population, which we believe to be the first study of its kind. Fermentations conducted in the winery had fewer unique strains than expected and were significantly higher in commercial strain abundance at the end of fermentation, Commercial strains were significantly more abundant in winery fermentations than in the vineyard equivalents, demonstrating that the winery environment significantly alters initial *S*. *cerevisiae* population composition. Interestingly, the average relative abundance of commercial strains in winery fermentations is lower (31.9%, 63.9% including commercial-related strains; Table 3) than those found in the two other studies of Canadian Pinot Noir spontaneous fermentations, where commercial strains represented at least 79% of all *S*. *cerevisiae* isolated [14,16]. The high abundance and diversity of commercial-related strains in winery fermentations (Fig 3, Tables 2 and 3) were not expected, and they suggest that a population of yeast descended from commercial strains is resident in the winery facility. *S*. *cerevisiae* found in wine-associated environments are typically diploid with some instances of aneuploidy [32,60]. While some commercial-related MLGs have heterozygous mutations due to losing or gaining microsatellite repeats at certain loci, most differ from their commercial counterparts by loss of heterozygous alleles; in some cases three or four per genotype (e.g. VL3). The loss of heterozygosity at these loci may be the result of gene conversion, where an allele is lost during double strand DNA repair processes; inbreeding and intratetrad mating; and in the case of complete loss of heterozygosity, haplo-selfing [58,61]. Variants of another commercial strain, VL1, recovered from vineyards in Portugal displayed distinct genetic and phenotypic differences from the parent strain that indicate adaptation to the vineyard environment [62,63]. In turn, our commercial-related genotypes may be evidence of strain adaptation to winery conditions after fermentation.

The appearance of commercial and commercial related MLGs in HE and HW block, which are approximately 700m distant from the winery, is a larger dispersal range for commercial *S*. *cerevisiae* than previously reported in the literature. In a study on vineyard-associated yeast in France and Portugal, Valero et al. (2005) found that commercial yeast were found almost exclusively within 100-200m from the winery facility, and mostly in post-harvest samples [19]. As some yeast found in the vineyards in our study were commercial-related or had not been used at the winery prior to sampling (e.g. Lalvin ICV GRE^®^, D254^®^), there may be resident commercial yeast in the vineyard as well, although their detection was inconsistent between vintages. A number of explanations may be behind the appearance of commercial strains in the vineyard and previously unused yeast strains in the vineyard and winery. Yeast are dispersed by a variety of mechanisms including through use of previously used equipment and barrels [21], insects [21,64], birds [65], and certainly humans. Considering that all three vineyards in our study are managed by the same staff and equipment, it is reasonable to assume that yeast could be transported by equipment and human contact. Lastly, there are at least two wineries operating within a 1km vicinity of SBV’s facility and vineyards and several more within a 3km radius, all of which may be possible sources of these strains. Whether these and other commercial strains could persist in the vineyards over a longer period requires further research.

### *S*. *cerevisiae* population structure and genetic differentiation

The low level of genetic differentiation (all F_ST_ < 0.06) in our dataset is understandable given the short distances and shared management among all vineyard locations. In their studies of yeast populations in winemaking regions of New Zealand, Gayevskiy and Goddard (2012) and Knight and Goddard (2015) derived F_ST_ values of 0.12 and 0.18 when comparing *S*. *cerevisiae* populations in commercial winery spontaneous fermentations and in vineyard soil and juice samples from regions 40-300km and 100-1000km apart, respectively [23,26]. Schuller et al. (2012) found somewhat higher differentiation in their study of vineyard-associated *S*. *cerevisiae* populations in Portugal between regions 180km apart [24]. The vineyard locations in our study are exponentially closer and thus subject to increased gene flow between populations through the mechanisms described above. One recent study profiled *S*. *cerevisiae* populations in winery fermentations from two closely situated estates (1.2km) and found an F_ST_ similar to our own [33]. Notably, the aforementioned studies identified few to no commercial yeast despite their use in these regions and even in adjacent fermentations at certain facilities [23]. In another New Zealand survey of winery-associated *S*. *cerevisiae*, none of the 88 genotypes shared more than 55% of their 18 typed alleles with commercial strains [21]. In contrast, commercial and commercial-related yeast account for over 100 of the 254 genotypes we detected, and they represent at least 5 of the 15 subpopulations in our Bayesian clustering results. Additionally, the removal of commercial and commercial-related genotypes from fixation index analyses universally reduced location F_ST_ values and rendered some values insignificant (Table 6). These results, along with the large contribution the winery population and VL3 subpopulation make to the overall ObStruct R^2^ value are evidence that commercial yeast use accounts for a substantial proportion of genetic differentiation between sites and is a factor driving population structure, along with location. The prevalence of commercial yeast in Okanagan spontaneous fermentations described in this study and in [14,16] appears high relative to other wine regions, even those of similar age (e.g. New Zealand), warranting more widespread investigation of British Columbian wine yeast populations.

Despite the prevalence of commercial and commercial-related MLGs and the level of genetic differentiation attributable to commercial strains, we did identify MLGs that appear unrelated to commercial strains, some of which were detected in all locations and persisted across vintages. The Bayesian clustering and phylogenetic analyses provide evidence that there are *S*. *cerevisiae* populations indigenous to SBV vineyards and winery. Identification and characterization of *S*. *cerevisiae* populations associated with wild ecosystems more distant from BC wineries and vineyards could provide definitive support to the existence of an indigenous BC *S*. *cerevisiae* population.

## Supporting Information

S1 Fig Genotype accumulation curve for SBV *S*. *cerevisiae* isolates. Boxplots represent random allele sampling with replacement (n = 1000) at each locus. The red dashed line denotes 90% of the total multi-locus genotypes identified in the dataset.
(TIFF)

S2 Fig SBV MLG frequency by Bruvo’s Distance to the nearest commercial strain. MLGs are binned by 0.05 Bruvo Distance units. The MLG density curve is represented in red.
(TIFF)

S1 Table MLGs identified from 1927 SBV isolates, 2013–2014. (XLSX)

S2 Table Fermentation sample point isolate and genotype information. (XLSX)

S3 Table Commercial and commercial-related *S*. *cerevisiae* MLG distribution by location and vintage. (PDF)

S4 Table Population information by locus. (XLSX)

S5 Table Results of ObStruct analysis on CLUMPP-optimized InStruct output. (XLSX)

S6 Table MLGs belonging to unique subpopulations K6-8. (XLSX)
