# Supplementary figures and images for: Impact of Commercial Strain Use on Saccharomyces cerevisiae Population Structure and Dynamics in Pinot Noir Vineyards and Spontaneous Fermentations of a Canadian Winery
Source: PLoS One. 2016 Aug 23;11(8):e0160259. doi: 10.1371/journal.pone.0160259 (PMC4995015; doi:10.1371/journal.pone.0160259)

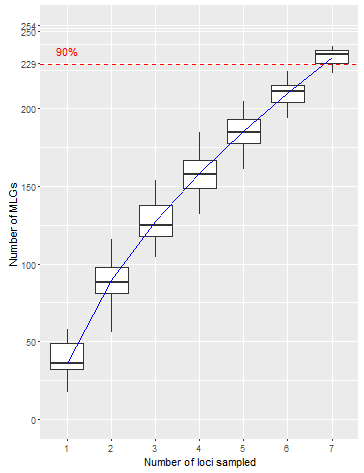

Supplement: S1 Fig — Boxplots represent random allele sampling with replacement (n = 1000) at each locus. The red dashed line denotes 90% of the total multi-locus genotypes identified in the dataset. (TIFF) [file pone.0160259.s001.tiff]

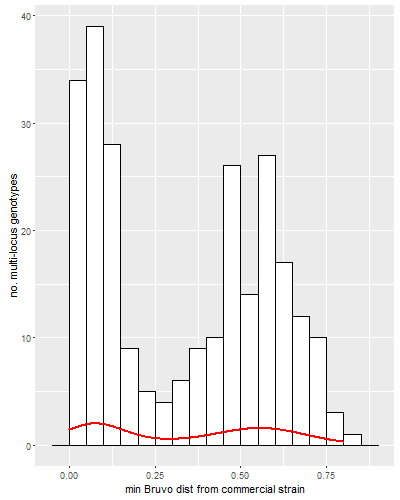

Supplement: S2 Fig — MLGs are binned by 0.05 Bruvo Distance units. The MLG density curve is represented in red. (TIFF) [file pone.0160259.s002.tiff]
