# Supplementary material for: Impact of Commercial Strain Use on Saccharomyces cerevisiae Population Structure and Dynamics in Pinot Noir Vineyards and Spontaneous Fermentations of a Canadian Winery
Source: PLoS One. 2016 Aug 23;11(8):e0160259. doi: 10.1371/journal.pone.0160259 (PMC4995015; doi:10.1371/journal.pone.0160259)
Supplement: S3 Table — (PDF) [file pone.0160259.s005.pdf]

S3 Table. Commercial and commercial-related *S. cerevisiae* MLG distribution by location and vintage.

MLG	No. MLGS	Winery		OG		HE		HW	
		2013	2014	2013	2014	2013	2014	2013	2014
Anchor VIN 13	1				Y				
Laffort Zymaflore® VL2	1	Y		Y		Y			
Laffort Zymaflore® VL3	1	Y	Y		Y			Y	
Laffort Zymaflore® FX10	1		Y		Y			Y	
Lalvin BA11®	1		Y						
Lalvin DV10™	1				Y				
Lalvin ICV-D254®	1	Y	Y	Y	Y	Y		Y	
Lalvin ICV-GRE®	1							Y	
Lalvin RA17® *	1		Y						
Lalvin Rhone 2056®*	1			Y					
Lalvin RC212 ®	1	Y	Y						
Uvaferm *S*VG™ *	1	Y	Y		Y	Y			
Vitilevure 3.001®	1		Y						
**Commercial MLG counts**	14	5	8	3	6	3	0	4	0
SB_522 Davis[#]	1							1	
SB_Anchor VIN 13	2				2	1			
SB_Anchor VIN 7[#]	1							1	
SB_Laffort VL1	1		1						
SB_Laffort VL2	14	2		5		11			
SB_Laffort VL3	40	14	28		3				
SB_Laffort X5[†]	3							3	
SB_Lalvin BRL97	2			1	1				
SB_Lalvin DV10	4	1			3				
SB_Lalvin ICV-D80[†]	4	3		2					
SB_Lalvin ICV-D254	2		1					1	
SB_Lalvin EC1118[†]	2	1			1				
SB_Lalvin ICV-GRE	5	1						5	
SB_Lalvin RA17[#]	4		4						
SB_Lalvin RC212	10	9	4					1	
SB_Lalvin R-HST[#]	1	1							
SB_Lalvin Rhone 2226[†]	2		1		1				
SB_Uvaferm SVG[#]	4				1	3			
**Commercial-related MLG counts**	102	32	39	8	12	15	0	12	0

Commercial-related MLGs are "SB" ("Stoneboat") followed by the name of the commercial yeast genotype they are most closely related to. Cells marked with 'Y' denote a commercial yeast was isolated was isolated from the corresponding location and vintage. Numbers indicate the number of genotypes isolated each location and vintage. OG, Orchard Grove Vineyard; HE, Home East Vineyard; HW, Home West Vineyard.

* Commercial yeast strains never used by the winery.

[#] Yeast MLGs related to commercial strains never used by the winery.

[†] Commercial-related MLGs whose parental strains were used in the winery but undetected in this study.
